# Binary Restrictive Threshold Method for Item Exposure Control in Cognitive Diagnostic Computerized Adaptive Testing

**DOI:** 10.3389/fpsyg.2021.517155

**Published:** 2021-08-05

**Authors:** Xiaojian Sun, Yizhu Gao, Tao Xin, Naiqing Song

**Affiliations:** ^1^School of Mathematics and Statistics, Southwest University, Chongqing, China; ^2^Southwest University Branch, Collaborative Innovation Center of Assessment for Basic Education Quality, Chongqing, China; ^3^Faculty of Education, University of Alberta, Edmonton, AB, Canada; ^4^Collaborative Innovation Center of Assessment for Basic Education Quality, Beijing Normal University, Beijing, China

**Keywords:** cognitive diagnostic computerized adaptive testing, measurement accuracy, item exposure rate, binary searching algorithm, cognitive diagnostic assessment

## Abstract

Although classification accuracy is a critical issue in cognitive diagnostic computerized adaptive testing, attention has increasingly shifted to item exposure control to ensure test security. In this study, we developed the binary restrictive threshold (BRT) method to balance measurement accuracy and item exposure. In addition, a simulation study was conducted to evaluate its performance. The results indicated that the BRT method performed better than the restrictive progressive (RP) and stratified dynamic binary searching (SDBS) approaches but worse than the restrictive threshold (RT) method in terms of classification accuracy. With respect to item exposure control, the BRT method exhibited noticeably stronger performance compared with the RT method, even though its performance was not as high as that of the RP and SDBS methods.

## Introduction

Cognitive diagnostic computerized adaptive testing (CD-CAT; Cheng, 2009, 2010; Chang, 2015) has attracted the attention of numerous researchers and educators over the past few decades (Wang et al., 2012). CD-CAT is a combination of a cognitive diagnostic model (CDM) and computerized adaptive testing (CAT). A key advantage of CD-CAT is that educators can provide remedial instruction for individuals based on the knowledge level of the individuals, which is determined using CDM (e.g., Gierl et al., 2007). In addition, CD-CAT can generate a test tailored to suit an individual's latent trait levels (Mao and Xin, 2013; Chang, 2015; Lin and Chang, 2019). Consequently, the estimation of an individual's latent ability is more accurate when fewer items are used compared with using traditional paper and pencil tests (Weiss, 1982).

One of the major objectives of CD-CAT is to improve classification accuracy. Numerous item selection methods have been developed to achieve this objective. Item selection methods commonly applied including the Kullback–Leibler method (KL; Xu et al., 2003), Shannon entropy method (SHE; Tatsuoka and Ferguson, 2003), posterior weighted KL method (PWKL; Cheng, 2009), and modified PWKL (MPWKL; Kaplan et al., 2015). Several attempts have been made to develop item selection methods for short-length tests, such as the mutual information (MI; Wang, 2013), posterior weighted CDM discrimination index, and posterior weighted CDM attribute-level CDI (PWACDI) (Zheng and Chang, 2016). All of the aforementioned item selection methods noticeably enhance the classification accuracy of CD-CAT. However, a major attribute of such methods is that they focus largely on maximizing classification accuracy rather than on controlling item exposure, which results in a highly uneven distribution of item bank usage. Although CD-CAT is used mainly for low-risk tests (Leighton and Gierl, 2007; Wang et al., 2011; Mao and Xin, 2013; Lin and Chang, 2019), where item exposure is not a major concern, items may be at risk of overexposure if an individual already knows the items before taking the test (Wang et al., 2011; Mao and Xin, 2013). In addition, it is not appropriate to administer an item bank with a large number of underexposed items because item bank development is a time- and money-consuming process (Wang et al., 2011; Zheng and Wang, 2017). To establish a balance between classification accuracy and item exposure control, several novel item selection methods have been proposed (e.g., Wang et al., 2011; Hsu et al., 2013; Zheng and Wang, 2017).

Wang et al. (2011) proposed the combination of the restrictive progressive (RP) and restrictive threshold (RT) methods with the PWKL method to achieve item exposure control for fixed-length tests in CD-CAT. In addition, Hsu et al. (2013) developed the Sympson-Hetter method and considers test overlap control, variable length, online update, and restricted maximum information (SHTVOR) to address item exposure with varied test length in CD-CAT. Recently, Zheng and Wang (2017) applied the binary searching algorithm for item exposure control in CD-CAT. They proposed the dynamic binary searching method for varied-length tests and the stratified dynamic binary searching (SDBS) method for fixed-length tests. However, even though the RP method could generate a more even distribution of item usage for fixed-length tests, the classification accuracy was considerably decreased. In comparison, the RT method achieved higher classification accuracy but a more uneven distribution of item usage. The SDBS method is a promising one-item selection method in terms of the testing efficiency and distribution of item usage, but it has relatively low measurement accuracy and flexibility. In addition, the SDBS method does not take into account item parameters, which potentially resulting in wasted item information and low measurement accuracy. To address the shortcomings of the aforementioned methods for fixed-length CD-CAT, we propose a modified method inspired by Wang et al. (2011) and Zheng and Wang (2017). The new method—the binary restrictive threshold (BRT) method—integrates the binary searching algorithm into the RT method.

The remainder of this paper is organized as follows: First, two commonly used CDMs in CD-CAT, the deterministic input, noisy “and” gate (DINA) model (Junker and Sijtsma, 2001) and the reduced reparameterized unified model (RRUM; Hartz, 2002), are briefly introduced. Subsequently, four item control indices—RP, RT, SDBS, and BRT—are presented to illustrate how such indices balance the trade-off between classification accuracy and item exposure control. Afterward, we perform a simulation study to compare the performance of the novel item exposure index with that of the RP, RT, and SDBS methods. Finally, discussions and conclusions are based on the findings of the simulation study are provided.

## CDMs

### The DINA Model

The DINA model is one of the most commonly used CDMs in CD-CAT because of its simplicity and ease of explanation (e.g., Cheng, 2010; Chen et al., 2012). It classifies individuals into two classes for each item: those who master all attributes that the item measures and those who lack at least one attribute that the item involves. The DINA model can be expressed as

$$\begin{array}{l} P\left(Y_{ij}=1|\eta_{ij}\right)={\left(1-s_{j}\right)}^{\eta_{ij}}{g_{j}}^{1-\eta_{ij}},\\ {\;\eta }_{ij}=\prod_{k=1}^{K}{\left(\alpha_{ik}\right)}^{q_{jk}}, \end{array}$$

where *Y*_*ij*_ is the response of individual *i* to item *j*; η is the ideal response indicating whether an individual master all the required attributes of an item; *s* is the slip parameter; *g* is the guess parameter; *K* is the number of attributes; α_*ik*_ denotes the deficiency or mastery of the *k* attribute for individual *i*; and q_*jk*_ is the element of the *Q* matrix.

One limitation of the DINA model is that it cannot distinguish individuals who lack one attribute from those who lack more than one attribute that a specific item measures. By contrast, the RRUM allows the probabilities of different attribute mastery patterns to vary across items.

### The RRUM

The RRUM has attracted considerable attention in CD-CAT in recent years (e.g., Dai et al., 2016; Huebner et al., 2018). The item response function of the RRUM can be expressed as follows:

$$P\left(Y_{ij}=1|\;\alpha \text{​​}{\;}_{i}\right)\;=\;\pi_{j}^{*}\prod_{K=1}^{K}r_{jk}^{*}{\;}^{\left(1-\alpha_{ik}\right)q_{jk\;}{\;}_{,}}$$

where $\pi_{j}^{*}$, the baseline parameter, refers to the probability of correct response to item *j* when individuals have mastered all attributes that the item requires; $r_{jk}^{*}$, the penalty parameter, denotes the reduction in the probability of correct response to item *j* when an individual lacks attribute *k*.

## Item Exposure Control Indices in Fixed-Length CD-CAT

### The RP Method

Wang et al. (2011) developed this item exposure control index. Two components are included in this method: the restrictive component and the progressive component. The former imposes a restriction to make sure that the maximum exposure rate does not exceed a pre-defined value, *r*. The latter component adds a stochastic element to the item selection methods to avoid the frequent selection of items with the largest amount of information (Revuelta and Ponsoda, 1998). The RP method can be expressed as follows:

$$\begin{array}{l} RP\text{\_}info_{j}=\left(1-\exp_{j}\text{/}r\;\right)\left[\left(1-\left(x/J\;\right)\right)\times R_{j}+info_{j}\times \beta x/J\;\right], \end{array}$$

where exp_*j*_ is the exposure rate for item *j, x* is the number of items that have been administered, *J* is the test length, R_*j*_ is a random value that is generated from a uniform distribution *U*_(0, max(info_r__j_))_, where *info*_*j*_ refers to the corresponding information of item *j*, such as PWKL information, and *β* is the importance parameter that is used to adjust the relative importance of classification accuracy vs. the item exposure control issue. A lower value of *β* indicates that test security is more important than classification accuracy, and vice versa.

### The RT Method

This method is another item exposure control index that was developed by Wang et al. (2011). It also includes two components: a restrictive component and a threshold component that is applied to derive an information interval. Candidate items during each interval can be randomly administered to individuals. The information interval is defined as follows:

$$\begin{array}{l} RT\text{\_}info_{interval}=\left[\max\left(info_{j}\right)-\delta ,\max\left(info_{j}\right)\right],\\ \;\delta =\left[\max\left(info_{j}\right)-\min\left(info_{j}\right)\right]\times f\left(x\right),\\ \;f\left(x\right)={\left[1-\left(x/J\right)\right]}^{\beta }, \end{array}$$

where *δ* is the threshold parameter and *β* is the importance parameter that determines the width of the information interval. The higher the value of *β*, the narrower the information interval. The rest of the symbols have meanings similar to those in the RP method.

### The SDBS Method

Zheng and Wang (2017) developed the SDBS algorithm, which stratifies items on the basis of their discrimination index. This method was inspired by the α-stratification method that is commonly used in IRT-based CAT (Chang and Ying, 1999). The classical testing theory (CTT)–based item discrimination indices for the DINA model and the RRUM are (1 − *s*_*j*_ − *g*_*j*_) and $\left(\pi_{j}^{*}-\pi_{j}^{*}\prod_{k=1}^{k}r_{jk}^{*}{\;}^{qjk}\right)$, respectively (Rupp et al., 2010). The SDBS can be computed as follows:

$$\begin{array}{l} \;B_{j}^{m}=\left|\sum_{S_{jl}^{m}=1}p\left(\alpha_{l}|Y_{t-1}\right)-0.5\right|,\\ \;S_{jl}^{m}=\prod_{k=1}^{K}I\left(q_{jk}\leq \alpha_{lk}\right),\\ p\left(\alpha_{l}|Y_{t-1}\right)=\frac{P\left(Y_{j-1}|\;\alpha_{l}\right)\pi_{0}\left(\alpha_{l}\right)}{\sum_{c=1}^{2^{K}}P\left(Y_{j-1}|\alpha_{c}\right)\pi_{0}\left(\alpha_{c}\right)}, \end{array}$$

where *B*_*j*_ is the binary searching index; S_*jl*_ is the separation for item *j* and attribute profile *l*, where S_*jl*_ = 1 indicates that the attribute profile *l* possesses all the attributes that item *j* measures and S_*jl*_ = 0 otherwise; *m* represents the *m*^th^ stage; *p*(**α**_*l*_|**Y**_*t*−1_) is the posterior probability for the *l*^th^ attribute profile conditional on the first *t* – 1 item responses; ***Y***_*t*−1_, *P*(**Y**_*t*−1_|**α**_*l*_) is the joint probability of the first *t* – 1 items conditional on attribute profile α_*l*_; π_0_(**α**_*l*_) is the priori probability; and *I*(.) is the indicator function, which equals 1 when the expression in the brackets is true and equals 0 otherwise.

The SDBS method tends to select an item with a lower *B*_*j*_ value as the next item to be administered. Because only the q-vector of item *j* is used during the calculation of *B*_*j*_ and item parameters (e.g., slip and guess parameter) are not taken into consideration, items measuring similar or even different attribute profiles can obtain consistent estimations of *B*.

### The BRT Method

Inspired by Zheng and Wang (2017), the present study attempts to combine the binary searching algorithm with the RT method to develop a novel item exposure control method. In particular, the binary searching algorithm is applied first to obtain the candidate item set that has the lowest binary searching index, *B*. The RT method is then used to select items from the candidate item set.

Because the BRT method combines the binary searching algorithm with the RT method, we expected it to achieve lower classification accuracy but superior item exposure control compared with the RT method. In addition, we expected the BRT method to achieve higher classification accuracy compared with the SDBS and BRP methods because the RT method, which is involved in the BRT method, can yield higher classification accuracy than the RP and SBDS methods (Wang et al., 2011; Zheng and Wang, 2017) when applied to select the appropriate item to be administered.

In the BRT method, items with the lowest B_*j*_ value can be obtained first, and then the RT method is applied to randomly select the next item from these items with the lowest B_*j*_ value. The mathematical expression of the BRT can be defined as follows:

$$\begin{array}{l} BRT\_inf\;o_{interval}=\left[\max\left(info_{j}\right)-\delta ,\max\left(info_{j}\right)\right],\\ \;\delta =\left[\max\left(info_{j}\right)-\min\left(info_{j}\right)\right]\times f\left(x\right),\\ \;f\left(x\right)={\left[1-\left(x/J\;\right)\right]}^{\beta },\\ \;j\in \underset{\min\left(B_{j}\right)}{Q}, \end{array}$$

where $\underset{\min\left(B_{j}\right)}{Q}$ is the q-vector of an item set with the lowest *B*_*j*_ value.

The difference between the BRT and RT methods is that an additional component, the calculated *B*_*j*_ value of each item, is considered in the BRT method. According to Zheng and Wang (2017), the additional component can be used to control item exposure.

In summary, the steps of the BRT method are as follows:

**Step 1**. Randomly select an item from the item pool as the first item to be administered to individuals;
**Step 2**. Estimate each individual's attribute profile;
**Step 3**. Calculate the binary researching index *B*_*j*_ on the basis of the estimated attribute profile;
**Step 4**. Determine the candidate item set that has the lowest binary researching index;
**Step 5**. Calculate the BRT index and select the appropriate item as the next item to be administered.
**Step 6**. Repeat steps 2 to 5 until the terminal rule is satisfied.

## Simulation Study

### Simulation Design

We performed a simulation study to evaluate the performance of the BRT method and then compared the BRT method with other item exposure control methods that have been proposed in previous studies (Wang et al., 2011; Zheng and Wang, 2017). In the present study, we manipulated factors such as model type, test length, number of attributes, and item selection method. These factors were set as follows:

#### Model Type

We included two model types in the present study, namely the DINA model and RRUM, both of which are models commonly applied in CD-CAT.

#### Number of Attributes

We applied four and six attributes in the present study, both of which are number of attributes that are commonly applied in CD-CAT (e.g., Cheng, 2009, 2010; Mao and Xin, 2013; Dai et al., 2016; Kang et al., 2017; Huebner et al., 2018; Lin and Chang, 2019). For instance, Wang et al. (2011) adopted four attributes in a simulation study, and Zheng and Wang (2017) applied four and six attributes in their study.

#### Test Length

There are two levels with respect to test length: 25 items (short length) and 40 items (long length). This setting is consistent with those applied in related studies (e.g., Wang et al., 2011; Zheng and Wang, 2017).

#### Item Selection Method

Six item selection methods—the random, original PWKL, RP, RT, SDBS, and BRT methods—were used in the current study.

The number of conditions was 2 (model type) × 2 (test length) × 2 (number of attributes) × 6 (item selection method) = 48 in total, among which only the item selection method was a within-group variable; the rest were between-group variables. The simulation study was implemented in R software (R Core Team, 2019), and the codes are available upon request from the corresponding author.

#### Item Bank and Examinees Generation

Two different item banks were generated on the basis of the number of attributes. Each item bank had 480 items, which is also a setting that related studies have commonly applied (Wang et al., 2011; Zheng and Wang, 2017). The item bank can be represented using the *Q* matrix, which describes the relationship between items and attributes. That is, the element of the *Q* matrix is 1 if the item measures the attribute, and the element is 0 otherwise. In the present study, the *Q* matrix was generated entry-by-entry conditional on independence among attributes. In addition, we assumed that each item involved at least one attribute and measured 20% of the attributes on average, which is similar to the case in Zheng and Wang (2017) study.

As for the item parameters, both the guessing and the slipping parameters of the DINA model were generated from a uniform distribution, *U*(0.05, 0.25). The baseline and penalty parameters of the RRUM were generated from *U*(0.75, 0.95) and *U*(0.20, 0.95), respectively. Other studies have also adopted such settings (e.g., Cheng, 2010; Wang et al., 2011; Chen et al., 2012; Mao and Xin, 2013; Zheng and Wang, 2017).

Two **α** matrices were generated to represent examinees' mastery of the attributes. Two groups of examinees were simulated, and each group was composed of 2,000 examinees. Similar to the *Q* matrix, the element of the **α** matrix was marked as 1 if examinees mastered the attribute and marked as 0 otherwise. The steps for generating the **α** matrix followed those proposed by Wang et al. (2011), and both the threshold and covariance among attributes were set as 0. Consistent with other studies (e.g., Cheng, 2010; Wang et al., 2011; Chen et al., 2012; Mao and Xin, 2013; Wang, 2013; Kaplan et al., 2015; Zheng and Wang, 2017), only one replication was used in the present study.

The value of the importance parameter (i.e., *β*) for the RT and RP methods was set to be 2. This is because Wang and her colleagues found that the value 2 can generate a reasonable trade-off between measurement accuracy and item usage (Wang et al., 2011). In regard to the BRT method, the value of the importance parameter was determined by a pilot study with varying *β* values. Its result showed that the value 0.5 is sufficient to balance the trade-off between measurement accuracy and item usage. Thus, the value 0.5 was selected for the BRT method in the current study. In addition, a total of five strata with equal number of items were used for the SDBS method, which recommended by Zheng and Wang (2017).

#### Evaluation Criteria

Two types of evaluation criteria were used in the current study. The first one was correct classification rate, which includes pattern correct classification rate (PCCR) and attribute correct classification rate (ACCR). Higher values indicate better PCCR and ACCR. The second criteria were item exposure control, which includes the scaled χ^2^ (Chang and Ying, 1999), number of items <2% (underused item rate; UIR) and more than 20% (overused item rate; OIR), and the test overlap rate (TOR; Mao and Xin, 2013). Lower values indicated favorable four item exposure control indices. The calculation of such evaluation criteria was performed as follows:

$$\begin{array}{l} \;PCCR=\overset{N}{\underset{i=1}{\sum }}I\left({\hat{\alpha }}_{i}=\alpha_{i}\right)/N,\\ ACCR_{k}=\overset{N}{\underset{i=1}{\sum }}I\left({\hat{\alpha }}_{ik}=\alpha_{ik}\right)/N,\\ {\;\chi }^{2}=\sum_{j=1}^{N_{item}}{\left(er_{j}-J/N_{item}\right)}^{2}/\left(J/N_{item}\right),\\ \;er_{j}=\frac{N_{j}^{administered}}{N},\\ \;TOR=\frac{\sum_{j=1}^{N_{item}}N_{j}^{administered}\times \left(N_{j}^{administered}-1\right)}{J\times N\times \left(N-1\right)}, \end{array}$$

where ${\hat{\alpha }}_{i}$ and α_*i*_ denote the estimated and true attribute profiles of examinee *i, N* is the number of individuals, *J* denotes the test length, *N*_*item*_ is the number of items in the item bank, $N_{j}^{administered}$ is the number of times the *j*^th^ item is administered (i.e., the number of individuals who answer item *j*), and *er*_*j*_ is the exposure rate of item *j*.

## Results

### Correct Classification Rate

Table 1 presents the correct classification rates for four attributes. The original PWKL and the random methods yielded the highest and lowest PCCRs, respectively, regardless of model type and test length. The RT method yielded the same or slightly lower PCCRs compared with the PWKL method and much higher PCCRs than the RP, SDBS, and BRT methods. In particular, the differences between the RT method and the three item exposure control methods (RP, SDBS, and BRT) were relatively small with respect to the DINA model, which ranged from 0.003 to 0.007 for 25 items, and no difference existed among the methods for 40 items. However, the differences were slightly greater for the RRUM, which ranged from 0.055 to 0.214 and from 0.008 to 0.052 for 25 and 40 items, respectively. The novel BRT method yielded slightly lower PCCRs than did the RT method, while it yielded higher PCCRs than did the RP and SDBS methods. The differences between the BRT method and the other methods (i.e., the RP and SDBS methods) ranged from 0.000 to 0.004 for 25 items, and they shared similar PCCRs for 40 items conditional on the DINA model. The differences ranged from 0.034 to 0.159 and from 0.036 to 0.088, for 25 and 40 items, respectively, conditional on the RRUM. In addition, among the four item exposure control methods (RP, RT, SDBS, and BRT), the SDBS yielded the lowest PCCRs under all conditions except one (*J* = 40, the DINA model). Regarding ACCR, the averaged ACCRs for PWKL and random methods were the greatest and lowest, respectively. The BRT method yielded slightly lower average ACCRs than did the PWKL but slightly higher average ACCRs than the RP and SDBS methods. In addition, the SDBS yielded the lowest average ACCRs among the four item exposure control methods.

**Table 1:** The correct classification for four attributes.

**Item selection method**	***J*** **=** **25**					***J*** **=** **40**				
	**PCCR**	**ACCR**				**PCCR**	**ACCR**			
		**A1**	**A2**	**A3**	**A4**		**A1**	**A2**	**A3**	**A4**
**DINA**										
PWKL	1.000	1.000	1.000	1.000	1.000	1.000	1.000	1.000	1.000	1.000
RP	0.997	0.999	1.00	1.000	0.998	1.000	1.000	1.000	1.000	1.000
RT	1.000	1.000	1.000	1.000	1.000	1.000	1.000	1.000	1.000	1.000
SDBS	0.993	0.998	0.998	0.997	0.998	1.000	1.000	1.000	1.000	1.000
BRT	0.997	0.997	1.000	1.000	0.999	1.000	1.000	1.000	1.000	1.000
Random	0.876	0.952	0.978	0.970	0.964	0.962	0.990	0.992	0.994	0.985
**RRUM**										
PWKL	0.986	0.998	0.994	0.996	0.996	0.999	1.000	1.000	1.000	1.000
RP	0.886	0.972	0.962	0.965	0.968	0.954	0.991	0.986	0.986	0.986
RT	0.975	0.994	0.992	0.994	0.994	0.998	1.000	1.000	0.999	1.000
SDBS	0.761	0.932	0.930	0.930	0.928	0.902	0.975	0.972	0.970	0.969
BRT	0.920	0.978	0.976	0.978	0.976	0.990	0.996	0.998	0.996	0.999
Random	0.572	0.865	0.874	0.865	0.864	0.716	0.913	0.930	0.923	0.904

*DINA refers to the deterministic input, noisy “and” gate model; RRUM refers to the reduced reparametrized unified model; PWKL refers to the posterior weighted Kullback-Leibler; RP refers to the restrictive progressive method; RT refers to the restrictive threshold method; SDBS refers to the stratified dynamic binary searching method; BRT refers to the binary RT method; PCCR refers the pattern correct classification rate; and the ACCR refers to the attribute correct classification rate*.

The PCCR results for six attributes revealed similar patterns with those for four attributes: the PWKL and random methods yielded the highest and lowest PCCRs, respectively, regardless of model type and test length. As for the remaining four item selection methods, their PCCRs are illustrated in Table 2. The RT method yielded the highest PCCRs under all conditions across the four methods. Furthermore, the BRT method yielded lower PCCRs than did the RT method; however, it had higher PCCRs than the RP and SDBS methods. The SDBS method yielded the lowest PCCR under all conditions. In addition, the differences in the PCCRs among the methods were relatively low for the DINA model compared with for the RRUM.

**Table 2:** The correct classification for six attributes.

**Item selection method**	***J*** **=** **25**							***J*** **=** **40**						
	**PCCR**	**ACCR**						**PCCR**	**ACCR**					
		**A1**	**A2**	**A3**	**A4**	**A5**	**A6**		**A1**	**A2**	**A3**	**A4**	**A5**	**A6**
**DINA**														
PWKL	0.995	0.999	0.998	0.998	1.000	1.000	0.999	1.000	1.000	1.000	1.000	1.000	1.000	1.000
RP	0.955	0.989	0.990	0.990	0.990	0.992	0.990	0.998	1.000	0.999	0.999	1.000	1.000	1.000
RT	0.992	0.997	0.998	0.999	1.000	0.998	0.998	1.000	1.000	1.000	1.000	1.000	1.000	1.000
SDBS	0.958	0.992	0.992	0.993	0.994	0.986	0.993	0.996	1.000	1.000	0.999	1.000	0.998	1.000
BRT	0.980	0.996	0.997	0.997	0.997	0.997	0.995	0.998	1.000	1.000	1.000	1.000	0.999	1.000
Random	0.650	0.942	0.930	0.896	0.931	0.918	0.924	0.829	0.972	0.968	0.966	0.971	0.966	0.960
**RRUM**														
PWKL	0.904	0.977	0.983	0.982	0.984	0.984	0.980	0.984	0.997	0.996	0.996	0.998	0.998	0.998
RP	0.705	0.944	0.938	0.921	0.940	0.940	0.926	0.847	0.978	0.972	0.964	0.974	0.968	0.958
RT	0.876	0.976	0.971	0.975	0.978	0.982	0.978	0.978	0.998	0.998	0.994	0.995	0.998	0.994
SDBS	0.542	0.908	0.896	0.880	0.896	0.900	0.892	0.772	0.962	0.950	0.956	0.952	0.950	0.948
BRT	0.726	0.946	0.940	0.937	0.939	0.943	0.958	0.907	0.986	0.979	0.978	0.981	0.987	0.985
Random	0.308	0.838	0.828	0.792	0.828	0.811	0.804	0.468	0.910	0.883	0.858	0.892	0.858	0.873

### Item Exposure Control

Table 3 presents the item exposure control for four attributes. The PWKL method had the highest scaled χ^2^ values, regardless of test length and model type, which indicated that the item exposure rate was quite skewed. In addition, the PWKL method had the highest TOR, UIR, and OIR values. For instance, more than 70% of the items were underused for the PWKL method, irrespective of test length and model type. In addition, the RT method yielded a slightly more even distribution of item usage than did the PWKL method, but it still had higher TORs, UIRs, and OIRs than the other methods, indicating that uneven distribution of item usage occurred. Compared with the RT method, the BRT method produced lower scaled χ^2^ values, TORs, UIRs, and OIRs under most conditions. That is, the scaled χ^2^ values that the BRT method produced were much lower than those produced by the PWKL and RT methods. The TORs were also lower than those of the RT under all conditions, and the UIRs of the BRT method were lower than those of the RT method under all conditions except one (*J* = 40, the DINA model). As for the RP and SDBS methods, the SDBS method yielded slightly better item exposure control than the RP method when the test length was short (*J* = 25); however, it performed slightly worse than the RP method when the test length was long (*J* = 40). Both the RP and SDBS methods performed better than the BRT method under all four indices (i.e., scaled χ^2^ value, TOR, UIR, and OIR). The differences between the BRT method and the RP and SDBS methods were relatively low in terms of the TOR and OIR and higher in terms of the scaled χ^2^ value and UIR. In summary, the BRT method yielded relatively poor item usage distribution compared with the RP and SDBS methods but more even distribution of item usage than the original PWKL and RT methods.

**Table 3:** The usage of items for four attributes.

	***J*** **=** **25**				***J*** **=** **40**			
	**χ2**	**TOR**	**UIR**	**OIR**	**χ2**	**TOR**	**UIR**	**OIR**
**DINA**														
PWKL	213.183	0.496	0.800	0.079	210.750	0.522	0.702	0.127
RP	5.116	0.062	0.000	0.000	2.475	0.088	0.000	0.000
RT	80.505	0.219	0.450	0.052	42.970	0.172	0.000	0.081
SDBS	3.949	0.060	0.010	0.000	4.488	0.092	0.000	0.000
BRT	12.837	0.078	0.208	0.000	9.604	0.103	0.027	0.010
Random	0.233	0.052	0.000	0.000	0.219	0.083	0.000	0.000
**RRUM**														
PWKL	225.213	0.521	0.825	0.079	228.810	0.560	0.752	0.142
RP	12.477	0.078	0.265	0.000	7.688	0.099	0.000	0.000
RT	145.923	0.356	0.665	0.075	167.067	0.431	0.569	0.135
SDBS	6.844	0.066	0.017	0.002	9.960	0.104	0.002	0.023
BRT	23.454	0.100	0.338	0.012	32.619	0.151	0.258	0.075
Random	0.220	0.052	0.000	0.000	0.215	0.083	0.000	0.000

Table 4 presents the results of item exposure control for six attributes. Most of the results in the table exhibit a similar pattern to that observed with four attributes. Specifically, the random method had optimal item exposure control under all conditions. As for the scaled χ^2^ value and TOR indices, the priority of the rest of the five methods was the RP, SDBS, BRT, RT, and PWKL. With respect to UIR and OIR, the RP method yielded the lowest values under all conditions except one (*J* = 25, the RRUM), in which the SDBS yielded the lowest UIR. The SDBS yielded lower UIRs and OIRs than the BRT and RT methods under all conditions, and the BRT method performed better than the RT method for the two indices under most conditions.

**Table 4:** The usage of items for six attributes.

	***J*** **=** **25**				***J*** **=** **40**			
	**χ2**	**TOR**	**UIR**	**OIR**	**χ2**	**TOR**	**UIR**	**OIR**
**DINA**														
PWKL	205.962	0.481	0.792	0.083	198.364	0.496	0.677	0.127
RP	4.952	0.062	0.000	0.000	2.272	0.088	0.000	0.000
RT	89.060	0.237	0.481	0.050	90.520	0.272	0.196	0.102
SDBS	9.333	0.071	0.190	0.000	10.053	0.104	0.010	0.021
BRT	20.152	0.094	0.298	0.008	21.490	0.128	0.144	0.050
Random	0.196	0.052	0.000	0.000	0.230	0.083	0.000	0.000
**RRUM**														
PWKL	199.284	0.467	0.783	0.092	200.328	0.500	0.690	0.135
RP	11.023	0.075	0.210	0.000	6.650	0.097	0.000	0.000
RT	122.366	0.307	0.638	0.075	143.225	0.381	0.548	0.125
SDBS	13.047	0.079	0.125	0.008	14.427	0.113	0.029	0.031
BRT	21.810	0.097	0.275	0.015	32.583	0.151	0.183	0.075
Random	0.225	0.052	0.000	0.000	0.205	0.083	0.000	0.000

## Discussion and Conclusions

Inspired by the studies of Wang et al. (2011) and Zheng and Wang (2017), we combined the binary searching algorithm with the RT method to develop the BRT method for CD-CAT. Because the core components of the SDBS method (i.e., a binary searching algorithm) and RT method were integrated into the BRT method, the RT method can be considered a specific case of BRT method, which means that the RT method can be obtained by adding some additional constraints to the BRT method. A simulation study was performed to investigate the performance of this novel item exposure control method. According to the results, the BRT method has more discernible merits than the PWKL and RT methods in terms of item exposure control, irrespective of the number of attributes, model type, and test length, although it yields slightly less accurate classification than the PWKL and RT methods under all conditions. The BRT method yields relatively poor item exposure control but more accurate classification under all conditions when compared with the RP and SDBS methods.

The results demonstrate that differences in the PCCRs between the BRT and RT approaches are minor for the DINA model, whereas the BRT method achieves superior item exposure control to the RT method. This is especially true when the scaled χ^2^ value and the TOR are examined. These findings indicate that the BRT method, to some degree, is a good candidate for the RT method when the DINA model is applied to CD-CAT with small number of attributes or long length of tests. Compared with the DINA model, the RRUM reveals larger differences in the PCCRs between the BRT and RT methods. That is, the RT method produces higher PCCRs than the BRT method in all conditions. However, the BRT method performs better than the RT method with regard to item exposure control. These results indicate that there is a trade-off between measure accuracy and item usage when a selection is made from the RT and BRT methods for the RRUM. As for how to choose reference values to interpret the evaluation criteria (e.g., scaled χ^2^, overlap rate), there are no definite answer, and reference values can be determined by test purpose. The BRT method can be used to select items to be administered if obtaining an even distribution of item usage is the primary goal, wherein both item exposure control indices (i.e., lower scaled χ^2^ and overlap rate) and measurement accuracy (i.e., higher PCCR) are important. In contrast, the RT method can be applied if measurement accuracy is the major consideration, such as classroom settings. In such situations, higher scaled χ^2^ and overlap rate are acceptable.

Furthermore, there might be a ceiling effect in measurement accuracy for the DINA model. This is because values of measurement accuracy are close to upper bound (i.e., 1.0) under both 25 and 40 items conditions. We further investigated this effect by conducting a pilot study with varying test length (10, 15, and 20 items) and varying number of attributes (four and six attributes) for the DINA model. Its results showed that the PCCRs are larger than 0.95 for the RT and BRT methods in conditions with 15 items and four attributes and are close to the upper bound in conditions with 20 items. The PCCRs are close to 0.95 under conditions with 20 items and six attributes and close to 1.0 under conditions with 25 items. These results confirmed the ceiling effect of measurement accuracy for the DINA model. In addition, the pilot study also showed that differences in the PCCRs between the RT and BRT methods are smaller for conditions with four attributes than those with six attributes regardless of which test length is used. In particular, differences in the PCCRs ranged from 0.0 to 0.02 and 0.0 to 0.09 for conditions with four and six attributes, respectively. This result indicated that the number of attributes has a positive effect on the differences of the PCCRs between the RT and BRT methods. The BRT method performs worse in PCCR than the RT method under conditions with large number of attributes and short length of tests.

Overall, the proposed BRT method, to some extent, can better balance the trade-off between correct classification and item exposure compared with prior methods. It yields slightly less accurate classification compared with the original PWKL and RT methods; however, it achieves superior item exposure control. In addition, although the BRT method provides slightly poorer item exposure control than do the RP and SDBS methods, it yields more accurate measurements.

Although the current study presents promising findings, the following potential future directions should be considered. First, the majority of studies that have explored item exposure have been based on the PWKL method. Other flexible methods, such as the SHE, MI, and MPWKL, should be investigated further. Second, both the DINA model and the RRUM are specific CDMs, which assume either conjunctive or disjunctive relationships between items in one tests. By contrast, the general CDMs relax the constraints of the specific CDMs. That is, they allow each item to select the optimal model to achieve optimal results (Ravand, 2016). Whether the new method can be applied to general CDMs is worthy of investigation in the future. It is worth noting that a variety of CDMs have been developed for varying situations in recent years, each of which makes specific assumptions about the relationship between item response and the attributes that item measured. Thus, assumptions that have been made for a situation, to some degree, determines the selection of a CDM. As well as data-driven model selection, for instance, use Akaike's information criterion and Bayesian information criterion to select the CDMs. Third, the application of the new method to the dual-objective CD-CAT (McGlohen and Chang, 2008; Wang et al., 2012, 2014; Dai et al., 2016; Kang et al., 2017; Zheng et al., 2018) could be investigated in the future. The dual-objective CD-CAT combines the IRT model and CDMs; therefore, it may be able to provide both an overall score and specific diagnostic information for individuals. Because the novel item exposure control method is proposed primarily for single-objective CD-CAT, it requires modification before application to dual-objective CD-CAT. Fourth, the new method could be extended to variable-length CD-CAT in the future study. However, it is important to note that the new method needs some modifications before its application to variable-length CD-CAT. This is because the posterior probability of an attribute profile is usually used as termination rule in variable-length CD-CAT. As such, the application of the new method to variable-length CD-CAT would be more complicated than its application to fixed-length CD-CAT. Last, true item parameters, rather than estimated item parameters, are used in the current study. As Huang (2018) and Sun et al. (2020) demonstrated, measurement accuracy is decreased when estimated item parameters are used. In other words, the reliability of item exposure control methods is relatively low with estimated item parameters. Therefore, further studies can consider investigating the reliability of the BRT method when estimated item parameters are used.

## Data Availability Statement

The datasets generated for this study are available on request to the corresponding author.

## Author Contributions

XS, TX, and NS proposed the original concept and designed the fundamental study of this study. XS and YG wrote the simulation study code and organized the article. All authors contributed to the manuscript revision.

## Conflict of Interest

The authors declare that the research was conducted in the absence of any commercial or financial relationships that could be construed as a potential conflict of interest.

## Publisher's Note

All claims expressed in this article are solely those of the authors and do not necessarily represent those of their affiliated organizations, or those of the publisher, the editors and the reviewers. Any product that may be evaluated in this article, or claim that may be made by its manufacturer, is not guaranteed or endorsed by the publisher.
